# Histoire de la Médecine depuis son Origine jusqu'au XIXe Siècle

**Published:** 1847-01

**Authors:** 


### ART. V.

1. *Histoire de la Médecine depuis son Origine jusqu'au XIXe Siècle.* Par le Docteur P. V. RENOUARD. Tom. ii.—*Paris,* 1846. 8vo.
2. *Saggio sopra il Modo e le Regole di osservare e massime in Medicina.* Del Dr. L. EMILIANI, Prof. Emer. di Clinica Medica e Medicina Pratica nella Regia Universita di Modena, &c.—*Modena,* 1844. 8vo, pp. 107.
3. *Zur Charakteristik der Medicin der Gegenwart.* Von Dr. J. M. LEUPOLDT, offentl. ord. Professor au der Universität zu Erlangen.—*Erlangen,* 1846. 8vo, pp. 93.
4. *The Medical Police of the United Kingdom.* From the Westminster Review for March. (A reprint.)—*London,* 1846.

THE student of M. Renouard's history may make much of it, if he be a dexterous reader, and have a vivid imagination. He may conjure up the dead, mighty in medical art and science; he may pass them before him as in a panorama. The shadowy forms of Egyptian priests will head the array; then will follow the scarcely less shadowy Greek priesthood, and the silent and contemplative disciples of Pythagoras. The Cnidians, and then the Asclepiades, marshalled by Hippocrates, will follow these; Socrates, Plato, and Aristotle mingling in their ranks. Then, once more, a long array of Egyptian sages will appear, the masters of the Alexandrian school, flanked by those of Pergamos, with a multitude of disciples, Pagan and Christian, Asiatic and Latin. Close upon these tread the Arabians, intermingled with Greeks of the declining empire; and misty forms bring up the rear, a motley commingled multitude. On they pass, until again Greek figures are visible, marshalling on the men of the dawning middle ages. Strange is their array! bearded and gowned necromancers, cowled priests, short-frocked bathmen and barbers, hooded women. Motley, too, their banners! The symbols of astrology and Christianity are written on them, with the names of Aristotle, Hippocrates, and Galen, of Plato, Avicenna, and Paul of Egina, of Celsus, Boëtius, and Cælius Aurelianus. But the light dawns more brightly as the panorama moves on, and Galileo, Bacon, Vesalius, and Harvey and Sydenham, and sages, whose names are as familiar to the medical student as his own, appear in the scene. They are intermingled, however, with Rosicrucians and alchemists, pretenders, and fantastic dreamers, amongst whom we recognize John Brown and Mesmer, Hahnemann, and our old friend Dickson; he of "chronothermalism," being both last and least.

The contemplation of such a *coup d'œil,* as this history of medical progress affords, cannot but instruct us. Who were the greatest? we ask, who the most worthless? how did the great attain to their fame? why were the unworthy worthless? when did medical art advance, and why? when did it recede? A consideration of questions like these must surely be of use to guide the ambitious or the noble of the profession, and to designate the false and frivolous. Those who fully appreciate the value of medical art to society, and rightly estimate the immense power slumbering in its practisers, may surely learn something from the history of two or three thousand years, and be better able to advance the one, or rouse and organize the other.

The profession and practice of medicine has no history anterior to the early Greek age. From the meagre details handed down by historians, it would appear that a perfect medical organization formed one of the elements of Egyptian society at the time Herodotus wrote; and medical art had its fixed rules, any innovation on which was penal. It would appear, too, that at a period, long anterior to Herodotus, the laws of hygiene formed part of the moral or religious code of Egypt, and probably of the nations of India and Central Asia. What influence these ancient doctrines had upon Greek literature is uncertain, but it must have been considerable, for it cannot be doubted that there was an extensive interchange of thought between the Greek and Egyptian priesthood, and in later times, between the Greek and Asiatic philosophers.

M. Renouard divides medical history into three great ages. The first includes the foundation of medical science, and extends from the mythological age to the time of Galen, or the termination of the second century of the Christian era. The second, which he terms the age of transition, includes the time between the end of the second century and the commencement of the fifteenth. The third is the age of renovation, and includes the time from the commencement of the fifteenth century to our own era. These ages are subdivided into periods or epochs. The first is the primitive period, or period of instinct, and ends at the fall of Troy, about twelve centuries before Christ. The second includes the mystic, or sacred period, and occupies from the fall of Troy to the dispersion of the Pythagoreans, 500 years before Christ. The third is named the philosophical period; it occupies about 180 years, and terminates with the foundation of the schools at Pergamos and Alexandria, 320 years before Christ. This school commenced the anatomical period, which ended with the second century after Christ. It was followed by the Greek period (the fifth), which continued until the destruction of the Alexandrian library, in 640. Science then passed over to the Arabians, and the Arabian period commenced, to end with the fourteenth century. With the revival of letters, medical science revived, and the erudite period began, which, so soon as the recovery of ancient literature was perfected, ended in the last, or reformation period, and which includes the seventeenth and eighteenth centuries. The first half of the nineteenth century belongs to the present period, and is at present left untouched by M. Renouard.

The progress of medical art and science, and of the medical mind, through those ages and periods, is typified in the progress of each individual student. At the outset, medicine was doubtless instinctive, although we are by no means inclined to place this outset at so late a period of the world's history as the fall of Troy. There is sufficient proof, we think, that it had grown to a science in the great Asiatic empires long before this comparatively modern epoch. But M. Renouard's notion applies accurately enough to European science. There is satisfactory evidence that two points only were involved in medical practice in Europe at this early period. The one, was a succinct description of diseases, or nosology; the other, an account of remedies suitable to the cure of the diseases described, or therapeutics.

With the progress of medical observation, it would doubtless soon be found, that a knowledge of healthy structure and function conduced materially to a better knowledge of nosology and therapeutics, and thus the sciences of anatomy and physiology would arise. The prevention of disease would also be an instinctive consideration, and hygiene would be developed. These, then, nosology, therapeutics, and hygiene, are the kernels of medical science. M. Renouard adds two other branches, orthopædy and phrenology, as completing medicine, which he defines to be the science that has for its object the preservation of health, the cure of disease, the physical perfection of mankind. A more comprehensive, but not a more concise, definition might be adopted than this; but it is sufficient for our present purpose, which is to inquire how this object can be best attained.

The works before us sufficiently indicate the agitation of the professional mind on this point. These examples taken from several such, mark the characteristics of the present epoch of medicine. They prove that the professional mind is bent upon a fuller development of medical science and practice than has been yet recorded in the world's history. Like straws on a deep and mighty current, they show its direction and course. The resolution is evidently taken, that there shall be a fuller investigation of theories and principles; a higher appreciation of the transcendent importance of medicine; a more systematic attempt to concentrate all human knowlege to work out its objects, regardless of prejudices and dogmas. We shall, therefore, take these works as text-books for our purpose, and proceed to draw such lessons of experience from the past, as may enable the profession to work out more successfully a nobler future.

Looking upon the panorama of medical history, we find it divided into two great periods, each in one degree the counterpart of the other. The one extends from the Greek origin of the science to the irruption of the Saracenic tribes, and is complete in itself, inasmuch as it comprises the first rise and extinction of medical science in Europe. Just as the knowlege of Asia and of Egypt was extinguished in passing over with empire to the Greeks, so the knowlege of the latter passed over to the victorious Arabians, and Europe sunk into barbaric darkness. This period continued until the age of renovation, which began with the fall of Constantinople, the immigration of learned Greeks into Italy, and the culminating point of Saracenic power in the fourteenth century. This second period is, however, as yet incomplete, inasmuch as the extreme point of development is not yet reached. In all other respects, it may be compared with the first. It had this advantage, however, that it commenced on a wider basis than its analogue. The accumulated knowlege from which the science of the first period was developed was imperfect, because the sciential records of the mystic age antecedent to it, were confined to an exclusive priesthood and a hieroglyphical language, and much, therefore, of Asiatic and Egyptian learning was lost to the Greeks. They began science almost anew, and transmitted their accumulated knowledge to their successors in a living language, and a language most perfect and rich. It was also expounded to them by men of acute and polished minds, who read and spoke the language in which it was written. This was an immense advantage; it was the advantage which the student with a master possesses over the student without a master. Nevertheless, the powers which guided the development of medical science in the two periods, the obstacles which arose, and the methods which were adopted to overcome them, were the same in both. We shall, therefore, move through them on two parallel lines, and not consecutively with M. Renouard. We shall, however, adopt some of our author's rules of procedure as set forth in the subjoined extract.

"Celebrated physicians influence the progress of their science and the value of their art, not by their writings only, but by their oral teaching, their character, and their conduct. Their lives present both models for imitation, and faults and stumbling-blocks to be avoided. Often also the primary education of a man, and the circumstances in which he is placed, explain the turn of his genius, and give the key to his successes or reverses. For all these reasons, I have not neglected to give biographical details regarding the most famous physicians, especially when those details have some connexion with the general history of the art, or include some question of morals.

"The sciences are not isolated from each other in their progress; they hold each other by the hand; they are usually simultaneous in their advance. An exception to this rule occurred, however, in the history of the human mind in Europe. During the middle ages dialectics and theology were cultivated with success, while other branches of human knowledge vegetated in a deep depression. But at the fourteenth century industry, the sciences, and the arts awoke from their long sleep. On the one hand, the civil and political organization of the nations of Europe became more perfect, and their material well-being advanced; on the other, the intellectual and moral faculties of individuals were developed. Mind took a higher, bolder, and better flight. The historian of medicine would, it appears to me, fail in one of his duties, if he did not take from time to time a general view of the social condition of the people..........

"Another fact extremely remarkable, and worthy of special notice, in a history of medical theories, is that they are all derived more or less directly from some system of philosophy. Consequently, we should have but an imperfect knowledge of these theories if we did not investigate their philosophical source. Nevertheless, too great an importance should not be attached to these analogies, nor should we profess to estimate the value of medical theories by them. It should not be forgotten that a system of philosophy may be false as a whole, and yet be true in its special application to medicine." (pp 10-11, vol. i.)

There is great profundity in these views, and at the same time they are of great value, as will be seen afterwards.

Proceeding then with our task, we pass over M. Renouard's account of medicine, as taught and practised by the ancient Egyptians and by the Chinese. The medical art of the Greeks, during the mythological and sacred period, corresponds in most respects to that practised in the middle ages. It was in the hands of kings, priests, and pretenders, and was apparently traditional. Such was medicine amongst the mass in Europe during the dark ages, and such it has been found in all semi-civilized people. Amongst the ancient Gauls the Druids were the philosophers, physicians, legislators, and priests. So also in Peru, Mexico, Florida, and Cuba, the priests were the astronomers, philosophers, and physicians, and were learned in both medical and physical science.

It was the sacerdotal order in primitive Greece, as well as in Europe during the dark ages, that practised medicine as an art, and gave rise to the medical profession. The mythology of the Greeks led, however, to an earlier separation of the practisers of the art from the great body of the priesthood. The science of medicine had its patron god, and the first

Greek temple erected to his worship was a temple at Titane, dedicated to Esculapius, about fifty years after the fall of Troy. His worship soon spread through the whole of Greece, and from thence into Asia, Africa, and Italy. The most celebrated temples of Esculapius were at Epidaurus, Pergamos, Cos, and Cyrene. The priests were termed Asclepiades; each temple was at once a sanatorium and a medical college; and the medical knowledge the priests possessed was strictly confined to the initiated. The temples were at once miraculous springs and watering-places; and the priests acted both on the body and mind of their patient. M. Renouard observes, that the temples of the god of medicine were generally situate in the most healthy localities; sometimes on the summit of a hill, or the slope of a mountain; sometimes on the coast, at some little distance from the sea, or near a thermal spring, or perennial fountain. Plantations of trees pleased the eye of the sick, and afforded them cool and solitary retreats, or broad and spacious glades. The sick and luxurious crowded to these temples as they now a-days visit Priessnitz or Scarborough. The fees of these physicians were the offerings of the sick to the god, and consequently it was of some importance to their pecuniary interests that the superstition and ignorance of their patients should be suitably acted on. Aristophanes very humorously shows us one of the methods used for this purpose. They had tame serpents attached to their establishments, of which the Greek comedian indicates the use. A valet loquitur, who had been under treatment:

"The priest of the temple of Esculapius, after having extinguished all the lights, told us to go to sleep, adding, that if any of us heard the hissing which indicated the advent of the god, we were by no means to stir. We therefore all remained in bed, and made no noise........As for myself I could not sleep, on account of the odour of a basin of excellent soup, which an old woman had near her, and which tickled my olfactories most amazingly. Being therefore extremely anxious to creep near her, I raised my head, and saw the sacristan take the cakes and figs from the sacred table, and going the round of the altars, put all that he found on them into a bag. It occurred to me that it would be very meritorious to follow his example, so I arose and went to grab the old woman's basin of soup."

We have made a rather free translation of this curious passage. Of course, visions, relics, and miracles were of daily use and occurrence. M. Renouard thinks the Asclepiades secretly took down a detailed history of each case, and of the means used for the cure, and that they tabulated these according to the seat of the disorder, beginning with the head, and so proceeding downwards. They were content to observe what remedies cured certain diseases, and to employ those remedies in similar cases; a principle at the foundation of all medication, but seen in its greatest purity in the practice of lay amateurs and quacks. Nothing is more common amongst laymen than the expression, "take such and such a remedy, it cured a case exactly like yours." The difference between the scientific practitioner and the amateur is, that by study and experience, the former is able to discriminate resemblances and differences. But this power of discrimination is of slow growth. As a general rule, he cannot experiment like the physical philosopher. Nature and chance present him with *his* experiments, but their elements are never the same. It is often a lengthened period before an analogous case again occurs, and when it does occur, it is almost impossible to separate the subject of his experiment (the sick person) from numerous disturbing causes. Hence the maxim of Hippocrates, the greatest of the Asclepiades, *art is long, life is short, opportunities rare, experience deceitful, judgment difficult.*

With the progress of society in Greece, a development of the intellect took place, and philosophy introduced new views both in theology and medicine. The reformation also began to dawn, and Pythagoras was both the Luther and Galileo of his age. We have not space for a summary of his doctrines, but we may shortly observe, that after a prolonged tour through the then existing academies and seats of learning in Asia, he returned to his own country, and became an itinerant preacher in Greece and Southern Italy of the opinions he had there imbibed. He preached the doctrine of two eternal uncreated essences, namely matter and mind; the unity of the Deity; the homogeneity of nature; its indefinite perfectibility, and a doctrine of development in animated nature closely analogous to that laid down in the 'Vestiges of the Natural History of Creation.' On this point we subjoin an extract from a commentary on his doctrines in the 'Nouveau Dictionnaire d'Histoire Naturelle:' "He could conceive a time when insects, shell-fish, or the impure reptile knew no superior in the universe, and was at the head of organized beings.... ....Who knows whether, in the eternal night of ages, the sceptre of the world will not pass from the hand of man to that of a being more perfect, and more worthy to bear it? Perhaps the negro race, now inferior, reigned at one time over the earth, before the white man was created. If Nature has necessarily granted empire to races which she has formed more and more perfect, why should she stop her progress now? Who shall place limits to her power? She is upheld by God only, and it is his powerful hand which guides her."

Pythagoras founded numerous secret societies in Greece (in accordance with the custom of the times), and especially in Magna Græcia. These societies soon exercised an important influence on the morals of society, and on the governmental policy of the states. But their success encouraged their members to become ambitious, and they entered actively into political agitation contrary to the advice of their founder. This conduct drew down upon them the vengeance of the authorities; the priests, too, whose superstitions they exposed, and whose power they undermined, thundered anathemas against them; and the ignorant and immoral hated them for the purity of their lives and the superiority of their knowledge. The consequence was, a general persecution and a complete dissolution of the societies during the lifetime of their founder. Have we no modern Pythagoreans to whom this result should not operate as a warning? Are there not philosophers in this our day who hold identical doctrines, and who are pressing imprudently on popular prejudices? We trust the enthusiastic spirits abroad will learn wisdom by this experience, and not bring those great truths of science, which ultimately will place religious truths in an impregnable position, in contact with the deeply-rooted and vulgar prejudices which with many are substituted for religion.

*The Periodeutic Physicians.* The dispersion of the Pythagorean societies led to the establishment of a true medical profession. Previously there had been only two legitimate classes of physicians, the priest-physicians and the gymnasiarchs (corresponding to the bathmen, barbers, and chirurgeons of the middle ages), who superintended the health of the athletæ. But when the Pythagoreans were scattered abroad, a new state of things arose. While some became teachers of philosophy in the schools, and revealed the sciential secrets of their order, others commenced the practice of medicine, and visited the sick at their homes, a practice which gave origin to their name. Empedocles, of Agrigentum, was one of the most famous of this new class. A formidable competition thus arose against the priest-physicians, and they were compelled to abandon their monopoly of knowledge, and enter into the arena of literary and professional warfare with the rest. The priests of Cnidos were the first to publish, and they were followed by those of Cos (the temple, or college in which Hippocrates was attached), who gave to the world the basis of the series of treatises attributed to Hippocrates. These writings have various degrees of value, and were written by various authors. Hippocrates himself is reported to have followed in some degree the example of Pythagoras, and to have made a sciential tour through the Greek cities of Europe and Asia. The essays which properly belong to him are decided by M. Renouard, after a careful investigation, to be the following: 1. Some of the Aphorisms. 2. De Morbis populariter grassantibus; Lib. i et iii. 3. De Victus ratione in Morbis Acutis. 4. De Aëre, Locis, et Aquis. 5. De Articulus. 6. De Fracturis. 7. Mochlicus. 8. Predictorum Liber.

The whole collection, as it exists at present, was not made until about the period when the great scientific libraries of Alexandria and Pergamos were founded. It is supposed that the relatives and descendants of Hippocrates wrote several of the treatises in the collection, and sent them forth with the sanction of his name.

Hippocrates had probably derived some part of his high moral principles from the Pythagoreans. His love of truth and of virtue, his patriotism, his unwearied benevolence and unflinching disinterestedness, have contributed to render his name immortal, quite as much as his scientific and practical knowledge. He was the type of the philosophic physician; he had attained to that depth of knowledge, that he knew his own ignorance. He was also the contemporary and friend of Plato and Aristotle. The prejudices of the age and people prevented the cultivation of anatomy; this prevented progress in physiology, and thus the advancement of medical science could only be effected by clinical observation, and theories deduced from the philosophical systems in vogue. The Hippocratic era corresponded to the 14th, 15th, and 16th centuries, as, in that period, the superstition of the priests prevented the general cultivation of anatomy. The philosophical systems were also founded on foreign lore brought in by Pythagoras and his contemporaries, just as the dialectics of the 15th and 16th centuries were founded on the philosophy of Plato and Aristotle, brought into Italy by the fugitive Greeks from Constantinople.

When Aristotle had exhausted the philosophy of his day, he turned his attention to the cultivation of natural history, especially comparative anatomy and physiology. The philosophical period was about to pass into the anatomical. Aristotle's father was a physician (probably a periodeutic), and died when his son was young; and he, having entered the army, and being a dissipated young fellow, soon spent his whole fortune. He then abandoned war for his early studies, philosophy and medicine; and attended Plato's lectures with the greatest assiduity. While a student, he was also a general practitioner, and kept an open shop. He was a Macedonian by birth, and had the good fortune to be selected by King Philip as tutor to his son Alexander; and when the latter proceeded to the conquest of Asia, Aristotle kept up a correspondence with him on matters of science, and advised with him as to the specimens in natural history that were the most desirable to be sent home. It is not probable that his was the first museum of natural history ever formed, but it must undoubtedly have been the most magnificent and extensive of the time. How interesting would the catalogue be at this day! The age of Aristotle corresponded to that of Vesalius, Fabricius, Eustachius, Servetus, &c. who lived at the end of the 15th and the commencement of the 16th century, and who were amongst the first to set systematically at defiance the prejudices against human dissections. Theophrastus followed Aristotle in phytology.

*The Anatomical Period.* The patronage which Alexander the Great bestowed upon the studies of his tutor was continued by his successors, but with increased vigour. Science now burst forth in her might. Eumenes in Mysia, and Ptolemy Lagus in Egypt, two of Alexander's generals, imitated their chief, and became rivals in scientific pursuits. The one founded the library and school of Alexandria, the other those of Pergamos. In a century after their foundation, the two libraries contained, according to report, above 800,000 volumes: of these, Pergamos possessed 200,000.

The rivalry between the two universities became so intense, that the kings of Egypt forbade the exportation of papyrus, hoping thereby to cripple the Mysian rival in the accumulation of books. This illiberal policy had fortunately a good effect, as it immediately led to the discovery of parchment as a substitute for the fragile papyrus.

Ptolemy Soter, and his son and successor Philadelphus, collected scientific men around them, arranged the Alexandrian library at a great expense, established an academy of science, or rather an university, and maintained a select corps of philosophers and teachers. Amongst these (maintained by Ptolemy Philadelphus), were the two distinguished anatomists, Herophilus and Erasistratus. The latter was reputed to be the grandson of Aristotle; he founded a medical school at Smyrna.

The whole cycle of science was cultivated by the Ptolemies. They fitted out commercial and geographical expeditions; they cultivated bibliography; they made extensive collections in botany and zoology. Medical science was, however, their favorite pursuit. They not only supplied their schools with subjects for dissection, but they dissected themselves. Such indeed, was the renown of the Alexandrian school, that, at the time of Galen, it was sufficient reputation for a physician to have studied there.

The foundations of correct anatomical knowledge were now laid, and much of the superstructure raised, although the anatomical school had but a comparatively short existence. But subtle questions in philosophy began to take the place of experiment and research; and, worse than that, the Romans conquered Egypt, and stopped human dissections. Julius Cæsar also destroyed the noble library, and although Cleopatra had that at Pergamos subsequently transmitted to Alexandria, her patronage was rendered in a great degree useless by the folly of the imbecile Caracalla, who slaughtered great numbers of the inhabitants, abolished the salaries of the professors, and put a stop to their meetings and common halls. The principal writers of the Alexandrian school that have come down to us are Galen, Aretæus, Cælius Aurelianus, Celsus, Pliny, Dioscorides, &c. Galen was the prince of his age, as Hippocrates was of the preceding. He was a native of Pergamos,—from time immemorial the seat of a medical college, founded originally by the Asclepiades, and, subsequently, the seat of the university which rivalled that of Alexandria. When he was already celebrated for his acquirements in the languages, history, mathematics, and physics, he was twice specially called by Apollo, in a dream, to practise medicine, and he, therefore submitted to the divine will. After a professional tour, in which he visited Alexandria, he returned to Pergamos, and was appointed surgeon to the circus, and attended the wounded gladiators. He then tried his fortune in the great metropolis, and with the greatest success, for he got into practice amongst the highest classes of society at Rome. His rapid progress, his disdainful pride, which he took no pains to conceal, and his fierce abuse of his contemporaries, raised him up a host of enemies. In his book 'De Prænotione' he accuses the physicians of his time of base jealousy and stupid ignorance, calls them scoundrels, poisoners, &c., and declares that, having unmasked them, he will leave a city where a man is esteemed only for the luxury and wealth he displays, and where unblushing charlatanism usurps the confidence of a stupid and frivolous public. Many worthy members of our profession have taken upon themselves the duty of "putting down" quackery. Vain hope! Sisyphæan labour! Fools there will ever be; fat and noble fools, as well as a vulgar herd, and all will have their appropriate vermin to prey on them, just as animals (entomologists tell us) carry about with them their suitable pediculi and cimices. It is a love of truth that leads us to war with quacks and quackery. We regret to see honest minds led into mazy error, and it is from a sentiment of duty to them that we hoist aloft our beacon. If they will heed the light, well; if not, we smile at and pity them.

Galen did not take the matter as coolly as he ought. He left Rome for some quiet little town, (perhaps his alma mater, and native Pergamos), "where," to use his own words, "every man knew his neighbour, his birth, education, fortune, conduct; and where, consequently, vulgar pretence and quackery could obtain no countenance." His value was already too well known, however, at Rome, and the Emperors Marcus Aurelius and Lucius Verus called him back; he had, also, the confidence of their successors, Commodus and Septimus Severus. He died in the seventy-first year of his age. The epoch of Galen may be compared with that of Boerhaave and Cullen: it was, however, the culminating point of ancient medicine. The arrest of anatomical research led inquirers to occupy their minds in the digestion of knowledge already attained, and in the formation of sects and theories. All was confusion, but there was little progress. We shall revert to the theories and systems of this age as admirably illustrative of those of our own.

This unprogressive age was distinguished towards its close by compilers. The principal of these were Oribasius, in the fourth century (a native of Pergamos); Aëtius at the beginning of the sixth, a student of Alexandria; Alexander Trallianus, a Lydian, the son of a physician, probably contemporary of the latter; and Paul of Egina, a student and probably a professor at Alexandria in the first half of the seventh century. The works of Paulus Ægineta constitute a complete cyclopædia of the medical literature of his day. He had scarcely passed from the scene, when the Saracens (A. D. 640) scattered the Alexandrian school, and its library of 500,000 volumes heated the public baths for six months. This catastrophe finished the Greek period. The Franks, Visigoths, and Lombards had already overrun civilized Europe, and the arts and sciences found protection only at Constantinople and with the Arabian caliphs. The following remarks on this period (with which the era of European medicine closed) are made by M. Renouard:

"At the time of Galen animals were still dissected; and this professor informs us that he used apes for his anatomical demonstrations, the structure of which closely resembled that of man. Sometimes, indeed, the army surgeons obtained permission to open the body of some barbarian which they had taken from the field of battle; but, ultimately, the practice of dissection ceased altogether, and the structure of the human body was studied only in books, the horror of the early Christians to cadaveric researches being greater than even that of the Roman Pagans, and the fathers of the primitive church launched their thunders against this violation of the mortal remains of man.

"This neglect of anatomy, without doubt, contributed much to the decline of medical art; but other causes co-operated not less powerfully. In the first rank must be placed the rapid spread of Christianity, which disorganized the Pagan schools, discredited the profane sciences, and ruined the teaching of them. It excited in all men an ardent desire for that religious controversy which created so much disorder in the rising church, and hastened, as is well known, the fall of the Eastern Empire. In the second place, the small number of those who continued to cultivate natural philosophy, encumbered by a vicious method, sought only for the explanation of phenomena in the works of the ancients, and, fearing to innovate, trod stupidly in the beaten path of the past. Only two men, Alexander Trallianus and Paulus Ægineta, during the lapse of four centuries, showed the least originality; the one adding a few observations to pathology and therapeutics, the other making some decided improvements in surgery." (Vol. i, p. 408.)

The Arabian period comes in like an interlude between the fall of ancient and the rise of modern European science. The Arab caliphs were enlightened rulers; it may be that the light of knowledge had shone upon them from Pergamos and Alexandria, and created in them a love of letters. Be this as it may, their courts were a refuge for the intellectual Christians, which each dominant sect persecuted from their country. Haroun al Raschid was the most celebrated of these. He established, at Bagdad, both hospitals and public schools. His successor made still greater efforts. The university of Bagdad, founded by him, was the most celebrated of the middle ages, and almost equalled that of Alexandria. He instructed his ambassadors to purchase medical and philosophical manuscripts at any price, and he employed a Christian for forty years to translate them into Arabic, paying him for his translations with, literally, their weight in gold. About the same period science flourished in Spain under the enlightened rule of the Moorish princes. The library of Cordova contained 224,000 volumes.

Rhazes is the first of the Arabian writers who appears in history; he flourished at the close of the ninth century, and the commencement of the tenth. He was a Persian by birth, and was one of the most distinguished professors at the university of Bagdad, and the chief physician of the great hospital. At the age of eighty years he ceased to practise, from the loss of sight. In one of his works, dedicated to the Caliph Almanzor, he gives some advice on the choice of a medical attendant, which we think worthy quotation.

"Ascertain with care the antecedents of the individual to whom you propose to intrust that which is dearest to you, namely, your health, your life, and the health and life of your wife and your children. If that individual wastes his time in frivolous pursuits or in parties of pleasure, or if he cultivates too curiously arts foreign to his profession, as music, or poetry, or if, especially, he be addicted to wine, beware how you intrust to such hands so precious a deposit. He only merits your confidence who has applied himself at an early age to the study of medicine, attended upon able masters, seen many sick, and joins personal observation to a diligent perusal of the best writers; for it is impossible to witness everything, or investigate everything for yourself. The knowledge and experience of a single individual, compared with the knowledge and experience of all men and of all ages, is like a small thread of water by the side of a mighty river." (Vol. ii, p. 414.)

Hali Abbas, a Persian, and Avicenna, born A.D. 980, at Bokhara, were nearly contemporaries. The latter studied medicine and philosophy at the university of Bagdad; Albucasis was a native of Cordova, and lived at the commencement of the 12th century. He, as well as the former, was a compiler, principally of the Greek authors. Neither of them dissected; and their anatomical knowledge was derived almost altogether from the works of Galen.

Greece and Italy during this period were not altogether without light. Actuarius (a term signifying a court physician) lived at Constantinople, about the end of the 13th century. He wrote a book, 'De Methodo Medendi,' in which he expounded the Galenic doctrines, and introduced some Arabian therapeutics. In Italy, the school of Salerno enjoyed a high reputation. It had a reputation as early as the 8th century, or before; some suppose, indeed, that it was founded by the professors who were driven from Alexandria by the Arabs in A.D. 640. It attained its greatest splendour between the 10th and 13th centuries. Constantine, of Carthage, surnamed Africanus, was one of its most distinguished professors at the end of the 11th century. Other remarkable physicians and surgeons of Western Europe were, Gerard of Cremona, who died in 1187; Guillaume de Salices, professor, first at Bologna, then at Verona, died in 1277-80; Arnauld de Villanova studied first at Paris, then at Montpellier, and afterwards in Spain, died in 1313; Lanfranc, a pupil of Guillaume de Salices, professor of surgery at Paris, in 1295; Jean Pitard, the reputed founder of the College St. Côme, in Paris (which previously, in 1311, was what our London College of Surgeons was a century ago, namely, a community of barber surgeons); and, lastly, Guy de Chauliac, a contemporary of the preceding.

We have brought our historical sketch down to the revival of medical science in Europe. The preceding authors and professors knew little except Arabic literature; the rays of Greek science came to them by a sort of double reflection. As the siege and sack of Alexandria led to the expulsion of the Alexandrian professors, and the foundation of the school of Salerno and of the Italian universities, so the siege and sack of Constantinople, in 1483, led to the revival of literature in Europe, by the expulsion of the learned Greeks collected in that city. These took refuge in Italy, and carried off with them all the manuscripts possible. Some years previously to their arrival, however, the study of Greek literature had commenced; as, in 1428, Nicolas Leonicenus, professor at Ferrara, translated the Aphorisms of Hippocrates, and some books of Galen, into Latin. Linacre, the founder of the London College of Physicians, was a pupil, at Florence, of Demetrius Chalcondyle, a Greek refugee, and translated some of the treatises of Galen. On his return, he founded a chair at Oxford, and another at Cambridge, for the expounding of the Hippocratic and Galenic works.

We need not retrace the ground we have already gone over. We have previously shown that this period is in all respects analogous to that in the ancient era, in which Pythagoras, followed by his disciples, by Plato, Aristotle, and Hippocrates, established the erudite school in Greece. That erudition led to dissection, the study of natural history, and natural philosophy, and ultimately to the foundation of the anatomical school in Alexandria. During the *renaissance* in Europe, dissections of lower animals only were practised; and it was not until A.D. 1482 that the University of Tubingen obtained permission from Sixtus IV to dissect the human body. From this period the prejudices against the practice began to soften down, and the modern anatomical school was founded.

Having thus marked the broad outlines of medical history, we will retrace our steps, and consider the theories, systems, and methods of treatment of the two great epochs, directing our principal attention, however, to that period of ancient medicine most nearly resembling our own, namely, the epoch subsequent to the anatomical period. We have seen that the light of science, amidst all the mutations of society, was never extinguished, but was handed down from generation to generation, that connected the past and the future with linked hands. We learn, also, that the influence of philosophical theories and systems, if once generally received into men's minds, never ceased to operate, and that the new were always connected with the old. We see, also, that there was a constant connexion between the theories of the day and the experimental researches of the day; and that the former were true and useful in proportion as the latter were numerous and profound. We shall see, too, that bold theories led to renewed researches, and renewed researches ended in renewed theories: a significant fact! and not to be passed over slightly by those who denounce all theory as useless.

*Origin of nosological systems and medical theories.* We have already observed (after M. Renouard) that the fundamental principle of therapeutics is founded on experience only—on the instinct to apply those means to the cure of diseases which have already been found useful in similar cases. Now, the first great difficulty in acting on this principle is to determine what cases are truly similar; and hence arose the necessity of accurate observation as the groundwork of accurate diagnosis. Without this, the physician was liable to continual mistakes, in considering cases as alike which were altogether dissimilar. This desire for more accurate diagnosis may be traced through every period of medical history, and has never been so impulsive, and so rich in results, as in the present. The discovery of aids to diagnostic research is, indeed, the chief characteristic of modern medicine; and here we beg to state our first requisite to improvement in medicine, namely, a further development and perfection of the instruments by which observation may be rendered more minute and more accurate. There is, doubtless, ample room for this. All the senses, and also the intellect, may be much more efficiently aided than they are at present.

The first effort at diagnosis was, doubtless, an elaborate classification of histories of cases, according to their phenomena. But as all the phenomena were at first mingled together, whether essential or not, the method led to confusion. This was pointedly noticed by Hippocrates, as the fault in the writings of the school of Cnidos. The necessity thus arose to distinguish the phenomena that were permanent or transient, essential or accessary, primary or secondary, &c.; and out of this arose discussions as to the nature and essence of diseases, their causes, symptoms, progress, &c. Thus medical theories arose concurrently with nosological systems, and the study of philosophy and physical science began. By this time society had advanced in Greece; a wealthy class was formed, who partly devoted their time to intellectual pursuits, or encouraged the fine arts; and knowledge was vigorously sought after. The theories of supernatural causes were extinct amongst the better classes. This same desire for knowledge which, in recent times, has brought enterprising and wealthy Hindoos and Parsees to Europe, and has induced them to send their children to Paris and London for education, nay, which has brought students to Great Britain from distant China, operated on the ancient Greeks; and the intelligent and wealthy penetrated into Asia, that they might learn the arts and sciences of the ancient and highly civilized communities spread over its surface, and flourishing in its magnificent cities. Pythagoras is the only one of those who has left his name to posterity; but it is certain that he was not the first, although perhaps the greatest; for he left the impress of his mind on the physical and metaphysical science of Greece. The influence of his philosophy may be distinctly traced in the medical theories of the Hippocratic writings. The doctrine of the harmony of numbers is seen in the doctrine of crises and of critical days; in the theory of the four elements and the four humours (derived from Plato), of two elements, and of unity or one element.

Plato was the modifier of previous doctrines. He was, as regards medicine, the parent of numerous metaphysical theories. He considered that everything was to be discovered by meditation and mental intuition—by the force of thought only. He attempted to apply the mathematical method (already so successful) of founding physical science on axioms, or general principles, and plunged into the wildest hypotheses. The treatise 'on Ancient Medicine,' in the Hippocratic writings, contains a critique (as M. Renouard thinks) on Plato's method, which was adopted by many physicians, and shows that it is vain, and could lead to no result. The author stands up distinctly for observation and experiment in medicine. He maintains that there is no need to know first the nature of man, how he was created, &c., but that it is sufficient for the physician to observe accurately what is injurious to man, or what is beneficial, and to study his relations to surrounding agents.

Aristotle was a reformer in doctrine, as well as his master. He opposed him. He established that maxim which has descended with unimpaired force to the present day, "Nihil est in intellectu quod non prius fuerit in sensu." And this he applied practically.

"When," he says (we quote M. Renouard's quotation), "from a great number of experimental ideas a general idea is deduced respecting a whole order of similar things, that constitutes art. To have the knowledge that a certain remedy has been useful to Callias, to Socrates, and to many others, that is experience; but to know such a remedy is useful to all the individuals of the same species attacked by a definite affection, for example, to all men troubled with phlegm, or with bile, or with burning fever, that is art."

But Aristotle's views were influenced by those of Plato his teacher, and his second principle is, "that the first ideas excited in our minds are general ideas," and thenceforward he treads the Platonic road. But M. Renouard very justly remarks, that Aristotle confounded vague, obscure, and indeterminate ideas with true general ideas. This we think an important point to notice. What is the first mental act in the investigation of a patient's case? Do we not instinctively form at once a general idea of its nature, deducing this idea from the history of the first few symptoms? When the patient complains that he has cough, expectoration, and difficulty in breathing, do we not at once form a general idea of thoracic disease? Yet this general idea manifestly differs from a general idea, principle, or fact in philosophy; for the one is vague, doubtful, and indeterminate, while the other is precise, certain, and definite. And it is to the former class of ideas that philosophical doubts are applicable. On this point Aristotle observes, "the men who wish to learn, must first know how to doubt, for science is nothing else than the solution of antecedent doubts; but he who knows not of the knot cannot untie it."

The philosophy *proper* to Aristotle, was that which was antagonistic to Plato's, and which Bacon, Locke, Hume, Condillac, and others among the moderns, have adopted. It was the doctrine of experiment and observation as the basis of science, and led to all the discoveries in anatomy and physiology of the Alexandrian school. Nevertheless, the spiritualism of Plato advanced on the same parallel with it, and was indeed fed by it; the one was not free from the other, and as facts, observations, and experiments accumulated, the spiritualists, whether experimenters with Aristotle or true Platonists, seized upon them, generalized them into vague, doubtful, and indeterminate principles, and established new theories and systems. And this was done even to the most modern times. Leibnitz, Descartes, and Kant are the most prominent amongst the moderns of this school, but inductive philosophers, as Sir I. Newton for example, often practised the method. We think, indeed, that spiritualism is as instructive in its history and origin, and as necessary to scientific progress as sensualism; its office is to raise in men's minds the philosophic doubts we have just alluded to, and urge on to further researches for their solutions; to tie the knots which can only be untied by some accurate and more extended researches. Thus TRUTH has to emerge, like the beautiful order of creation, from the action of continually antagonizing forces, and rise in majesty and glory from what appears utter confusion. Why then should scientific rixæ be frivolous, or foolish, or embittered? Let medical philosophers, at least, carry on the perpetual flux and reflux of opinions and facts, and of facts and opinions, with the same still grandeur and profound repose amidst unceasing motion, which characterize the forces of the universe.

We shall not trace in detail the progress of anatomical and physiological science, and of the art of healing, made during the anatomical period. It is sufficient to say, that when compared with the knowledge of the previous erudite period, the progress was comparatively as great in every branch of medicine, as that which has been made since the revival of letters and science in Europe, or even during the 1600 years which have elapsed since its decline. It was a wonderful step, and could only have been effected by high intellect, urged on by the regal munificence we have before mentioned. In proportion as experimental research declined, spiritualism or dogmatism became predominant, and divided philosophers and the profession into numerous sects. Some followed the old masters, and some the new, but others (the Eclectics) acknowleged no master whatever. Amongst the earliest leaders of the anatomical school, the metaphysical doctrines and theories of Hippocrates, Plato, Aristotle, and Praxagoras—the successor to Aristotle (whose pupil, indeed, he was)—held firm ground, and while they experimented, they vaguely generalized, and were lost in mazy hypotheses. The sects were then termed Hippocraticians, or Praxagoreans, or Herophilists, &c., just as in modern times, there were Aristotelians, Galenists, Animists, Brownists, &c. But these sects had in their turn antagonistic sects. Philenus of Cos, and Serapion of Alexandria, for example, attacked the fundamental doctrine of Herophilus and others (derived from the Greek masters), and asserted boldly, that the doctrines of elementary qualities, cardinal humours, essences of diseases, were all false and hypothetical. They even rejected that fundamental doctrine which their opponents had derived from the Greek medical philosophers, —*contraria contrariis curantur*. They confined themselves entirely to the results of experiment and observation, and were the determined opponents of all spiritualism, all *a priori* general ideas, all hypotheses. They were the founders probably of the sect of Empirics, and maintained views closely analogous to those expounded in the Hippocratic treatise on Ancient Medicine, already referred to as that in which the doctrines of Plato and his method of research are condemned. Medical science and skill were to be acquired, the Empirics said, by three modes. The first was, personal observation, or autopsy; the second, the study of observation recorded by others, or history; the third was, epilogism and analogism, and consisted in deductions drawn from autopsy and history, and enabled them to make discoveries, or led to further observation. The autopsy of the Empiric was made with the greatest care, that errors in observation might be avoided. It was necessary that a disease should have been carefully watched under every variety of complication and circumstance; and a method of treatment carefully followed in the same manner, before the autopsy of that disease was considered complete. In short, they appear to have used a numerical or inductive method. The practitioner who had thus carefully observed a disease, and preserved a perfect knowlege of its symptoms, course, termination, and treatment, had arrived at a theorem; and he who had accumulated a sufficient number of theorems, was an experienced or empirical physician.

Epilogism, or analogism of the Empirics, was the application of the experience of known diseases to unknown by means of resemblances or analogies; that is to say, a new disease was treated according to the method established for one to which it had the closest resemblance. And the same plan was adopted in substituting one remedy for another.

The definitions of the Empirics never included latent causes, or essential, elementary, or primary properties. They never defined fever, with Galen, to be a heat contrary to nature fixed in the heart, or, with Asclepiades of Bythinia, an acceleration of the motion of the blood, caused by obstruction of the pores; they simply said, fever is an affection which manifests itself by acceleration of the pulse, increased heat, and sometimes thirst. These definitions were termed hypotyposes. M. Renouard discusses the question, whence the rational or philosophical empiricism arose. Some authors, he observes, thinks that it was a deduction from the doctrine of the Sceptic or Pyrrhonian sect: Galen was one of these. We have observed, that the methods adopted by different philosophers have all a foundation in Nature. We generalize instinctively, but we observe instinctively, too, previously to the generalization. Plato adopted the process of generalization only, and thought truth was to be attained by the intellect alone. Aristotle adopted more decidedly than his master the other part of the mental process, namely, observation. But the knowlege attained by this process is never stationary; the process is continually repeated, and new observations and generalizations correct the preceding, and show their imperfection. This renders the truth of our generalizations doubtful, and the exclusive adoption of this part of the mental process (the scepticism) characterized the sect of the Pyrrhonians. Now it appears to us, that the Empirics adopted all the three stages of mental action into their philosophy; with Aristotle they observed, and inferred (autopsy and theorem); with Plato they generalized, and inferred (theorems and epilogisms); and with Pyrrho they doubted both the accuracy of their observations and of their generalizations. The doubts kept them within the bounds of common sense: in short, they appear to have been the truly practical men of their day; to have adopted the methods best calculated to attain to a true natural history of disease, and to improved therapeutics, and they may be taken as the best model for imitation by the moderns to be found amongst the ancient sects.

We quote the following interesting remarks by M. Renouard; their application to our own times is manifest:

"The circumstances amidst which the system of Empiricism was promulgated, were most favorable to its propagation. Medical theories had fallen, as we have seen, into confusion. All principles, all opinions, all methods were again subjected to inquiry. The recent anatomical discoveries, the introduction of a great number of new medicines, the properties of which were undetermined, the continually increasing rancour of philosophical disputes, had all shaken the ancient dogmas without substituting better, or any which commanded the general assent. Amidst such conjectures, a doctrine which promised to put an end to the continual changes of dogmatism, and to fix the barren uncertainty of scepticism, by relying entirely on the testimony of facts,—such a doctrine ought to have been received with enthusiasm, particularly by those practitioners whose daily experience demonstrated the uselessness of dialectics in the advancement of medical science."

Empiricism was active for a century and a half; it then fell, and its fall was complete. It was altogether banished for ages, and its name became a synonym for ignorance and pretension. But when it arose again in the systems of Bacon, Locke, and Condillac, it aimed at a universal dominion in science. M. Renouard very clearly shows some at least of the causes of its decline. The ancient Empirics did not go further than the construction of secondary generalizations; they did not arise to first principles or to definite axioms. They resembled in short, the builder who stops short in the erection of a great edifice. Such a doctrine could not satisfy the speculative temper of the ancient philosophers, and it was therefore neglected. We are inclined to think, however, that the cause of its decline may be more properly attributed to a failure in progressive observation. The cultivation of general, or descriptive, and pathological anatomy became impossible as Christianity spread, and physics were so imperfect, that no aids to observation seem to have been invented. There was thus an arrest of development in science, or rather a marasmus. No new matter was added to its system, and therefore it remained like a blighted creature. On the other hand, the scientific mind being turned from the observation and the accumulation of new facts, was perforce driven to expend its active energies on successive theories and systems, each being doubted and discarded almost as soon as set up.

Asclepiades of Bythinia established one of these systems; he founded, or led to the foundation of the sect of the Methodists. He went to Rome to teach rhetoric, and by his genius, talents, and tact, obtained the patronage and society of the most illustrious and aristocratic society in the metropolitan city. Not satisfied with this success in his proper profession, he turned doctor, and his ambition led him to aspire to be the founder of a new system and sect. The Epicurean philosophy being then the most fashionable at Rome, and being that which he had doubtless taught, he based his new system on the physical dogmas of Epicurus and Democritus respecting the atomic constitution of matter.

It is worthy note, that this has almost been the invariable influence of the doctrines of the day on medical science. During the climax of the erudite period of Europe, Fernel based his pathology upon the doctrines of Galen, which were then fashionable, and again established the therapeutic dogma (derived by Galen from Hippocrates) *contraria contrariis curantur*. The study of the occult sciences at the same period made their way into medical theories with Paracelsus. When these had flourished a while, a reaction took place, observations accumulated, and Galen and dogmatism gave way to Hippocrates and experimental philosophy. But of the latter numerous theories arose, not unmixed with antecedent ideas. Van Helmont's system included an "Archeus," derived from the occult sciences, and the doctrine of ferments derived from the science of chemistry, just emerging into day from the researches of the Rosycrucians and Alchemists. The theories of Sylvius, Willis, &c., were founded almost altogether on chemistry. The progress of mathematical and mechanical philosophy raised up a school of iatro-mathematicians, who attempted to reduce the problems of life to equations, and to explain vital action by mechanical theories. The circulation of the blood just then discovered and established by Harvey, also occupied a large space in all the theories of the day, but especially in that of Boërhaave. The hypothesis of Descartes led to the medical theories of Stahl; while Boërhaave, Van Swieten, and the later iatro-mathematicians, had Cullen and his contemporaries for their descendants. We thus arrive at another important lesson, namely, that the influence of prevalent theories is invariably exercised on those which displace them; this, therefore, should be carefully estimated and guarded against.

To return to Asclepiades. His theory was only a screen for hygienic practice. He was a Hahnemann without his infinitesimal doses, or the maxim "similia similibus curantur." His watchword was, to open the pores. Exercise, agreeable varied diet, wine, &c., was very pleasant treatment for the luxurious Roman, and as Asclepiades was a finished scholar and wit, and contemned and ridiculed all medical science and literature, he was very popular.

Themison was the disciple of Asclepiades; his doctrine differed, however; it was antagonistic,—analogous to that of Brown, with strictum and laxum for sthenia and asthenia. The treatment by the metasyncritical circle was peculiar to the sect of Methodists, an account of which is given by Cælius Aurelianus, and of which Themison was one of the founders. We have not space for the doctrines or therapeutics of this sect; it is enough to say, that they were so general and so simple, that one of the sect, Thessalius Trallianus, declared he could teach the whole of medical science in six months. Methodism had a close family resemblance through metasyncrisis to hydropathy, but a course of dietetics took the place of hydriatrics.

We must here close our notice of M. Renouard's work with a summary of the lessons which we have drawn from the history of medicine. We first find that therapeutics must be founded on experience. The method of treatment to be adopted in any particular case must be that which has most frequently been found effectual in some previous and analogous case. This experience, to be perfect, must be founded on an exact diagnosis, so that the analogy on which the treatment is based may be perfect. Exact diagnosis cannot be established without—1. An accurate observation of diseases, and a true record of their natural history. 2. A systematic arrangement and perfect generalization of the phenomena observed. These necessarily demand to be arranged on some principles or laws, and generalized according to some method. The latter must be the inductive method, the former the laws of vital action deduced by that method, or in other words, physiology; which in its largest meaning includes anatomy.

Theories, and vague, general ideas, or hypotheses, may be safely used to advance medical science and art. In establishing new remedies and modes of treatment, or in discovering the therapeutics of new diseases analogies may also be permitted, as instruments of progress. But in both instances a rational empiricism is carried out, and a philosophical scepticism should be practised as to the value and accuracy of the theories, hypotheses, and analogies thus adopted, and they should be tested in these particulars by new observations, and inductive generalizations. Thus then the foundation of all improvements in medical art and science, is observation.

It is, therefore, a question of vital importance to determine how observation may be best advanced and perfected. But, first, let us define the meaning of the term observation, and let us analyse the act of mind by which an observation is completed. The etymological meaning of the term implies more than an act of the senses; it means the careful investigation of a thing, and thus it includes an act of mind; for nothing can be investigated carefully without reference to its relations, whether they be the antecedents or coefficients. Consequently, the reasoning as well as the perceptive powers are involved in an observation.*

We become so apt at observation by practice, and the accumulated knowledge of science is so familiar to us, that the process of observation seems simple and intuitive. While feeling a patient's pulse we note its condition, and determine its relations to the heart, lungs, and other organs of the body, and to the general symptoms of the case, without any distinct consciousness of our existing knowledge, or that there was a time when these relations were not known—a time when the physician was ignorant of the structure and functions of the heart—when he knew not the difference between arteries and veins, or between the heart and the liver. The relations of the pulse were formerly, in fact, less known and understood, and the observation of the true value of its variations more difficult, than the microscopic relations of excretions are now. This conjunction of two distinct mental acts is strongly marked out by Professor Emiliani, as necessary to constitute a true scientific observation. First, a clear and precise conception of the thing to be observed; next, an examination of its reciprocal relations and connexions. He observes the one cannot be separated from the other, and the judicious combination of both mental acts is necessary to the progress of science.

The clear conception of a thing, and of its relations, implies, first, a proper perfection and use of the senses: a training or education of the senses is evidently necessary to attain this point, however perfect they may naturally be, just as the raw giant requires a pugilistic training before he can enter the ring. This training should not only develop the senses physically, but should systematically teach how the errors of sense may be avoided—a matter now left entirely to experience. It should also inculcate a thorough scientific knowledge of the aids to the senses which have been invented from time to time, as the stethoscope, &c., and of the principles on which they are constructed.

The examination of the relations and connexions of the thing observed (the second process in the art of observing) implies scientific knowledge. In medicine, anatomy, physiology, chemistry, &c., are accessory or collateral sciences, and are only valuable to the physician by extending his knowledge of these relations and connexions. When the stethoscope got into general use, it became absolutely necessary to determine more accurately than had heretofore been done the whole mechanism of cardiac and pulmonary action, so that the relations and true pathological value of the sounds communicated along that instrument to the ear should be properly

* Sprengel's definition of observation is analogous to this. He says: "Sequitur animadversionem *observatio*, quæ nihil aliud est, quam attentissima et studiosa succedentium sibi phænomenorum animadversio. Quæ ut in observationem transeat, oportet primum attendere animum ad res, earum formas, qualitates, et proprietates." (V. Sprengel, Inst. Med., vol. i, in introitu.)

estimated. The relations of the urinary secretion, and of the urinary salts, remain yet almost a sealed book. The first process of the act of observing is difficult, because it demands a refined chemical analysis for its completion; and the second is impossible, because we are yet far from an accurate knowledge of the vital processes by which the urinary constituents are formed in the organism, and eliminated by the kidneys, and know still less of their intimate relations and connexions with morbid phenomena.

If medical observation be considered in all its relations, whether theoretical or experimental, it will be at once manifest that no one individual can grasp the whole. No one can have the necessary perfection of *all* the senses—no one can possibly understand the application of *all* the aids to research—no one can be the walking encyclopædia of the medical sciences, and the miracle of medical knowledge, which should understand the relations of the whole cycle of observanda. A division of labour has been found practically necessary, as well in the scientific as in the social world. Hitherto, however, the division of labour has been almost fortuitous; anything like a system is of modern growth, and is to be found only in committees of investigation, which have arisen out of scientific societies. What is wanted in the medical profession is, that the whole body shall be organized, with a view to a systematie division of labour in practising the art, and advancing the science of medicine.

The author of the article in the 'Westminster Review' has attempted a sketch of such a systematic organization. Setting aside the plan for a union of the members of each "grade," in one or two central colleges, he proposes the assimilation of all grades in local colleges, for the purposes of self-government and scientific research. He argues that this principle of assimilation is already acted upon in the social unions of the profession; in the Medico-Chirurgical Society for example, and other societies of the metropolis; in the Provincial Medical Association, and other societies of the provinces; and that this principle is the only foundation for a perfect organization of the medical profession, because it has been found that, while all others have utterly failed, this only has been found to be attended with any degree of success, or can theoretically combine the different branches into one body. It certainly is necessary that the principle of association and systematic division of labour should be better carried out into practice, and local incorporations of the profession would be a good and substantial basis for the purpose.

The formation of therapeutical theories, and the observation of the action of remedial agents, are closely connected, both with each other, and with physiological and metaphysical theories. That theories and hypotheses are necessary to the progress of medical science and art we have already shown. The advancement of the curative art by analogies, or, in other words, the application of a method of treatment found by experience to be useful in one form of disease to another analogous form, is based on theory, or, at least, on a theoretical inference, which has to be tested or modified by careful experiment. It is known, however, that the human mind, in the mass, advances by oscillations between antagonistic theories and systems; and that the old are continually reproduced as new, to combat the old revived. It is necessary, therefore, that this continual recurrence to past theories be restricted within its proper limits; and for this purpose we think the measure recommended by Professor Leupoldt is eminently suitable, namely, the establishment of a chair of medical history and theory in every school. Professor Leupoldt proposes that the course should be divided into two parts, and occupy two sessions. The first part to include biology, anthropology, and hygiene; the other, general pathology, therapeutics, and the history of medicine. We are certain that some such course would be beneficial, inasmuch as it would fortify the student's mind against a too ready reception of any theory, or a bigoted adherence to any. He would estimate them at their proper value as subsidiary, and not essential, to the progressive advancement of medicine: as the means and not the end. He would be prevented fixing his faith upon any one theory, or system, exclusively, whether physiological or therapeutical. He would not be a medical sceptic, and indifferent to all opinions and systems alike; for he would learn that truth is in all—is as omnipresent in human knowledge as the great God of Truth is in His creation. Thus the student would be trained to be a true Eclectic, and seek for the truth in every system and every theory. He would find it in allopathic, hydropathic, or homœopathic systems; in bio-chemical, dynamical, or histological theories.

In conclusion. Whatever may be the result of the attempt now making throughout the civilized world to thoroughly reform medical science and art, we feel certain that the opinion enounced by Bacon, with reference to science in general in his day, and which is quoted by two of the writers before us, must be the watchword of the men of progress:

*Instauratio facienda est* AB IMIS FUNDAMENTIS, *nisi libeat perpetuo circumvolvi in orbem, cum exili et quasi contemnendo progressu.*

---

